# Brain Rest and Sleep

**Published:** 1892-06

**Authors:** 


### Brain Rest and Sleep.

The tendency of the present time, especially in our own country, is more than at any other time in the history of our country, to mental overwork and exhaustion of the brain forces.

The circumstances surrounding active professional life of to-day are such that the nervous system must be of the most perfect character to resist the immense strain made upon it. When one observes the influences brought to bear upon one in active city business, it is a source of surprise that there are not more cases of insanity from brain exhaustion. The demand of the time is for a far greater amount of brain rest, for longer periods from business, confusion, excitement, and the care, anxiety and responsibility of everyday business. In addition to the hours assigned to one's daily employment, there are engagements made, which seem essential, which encroach so largely on one's sleep, that when we are brought to think carefully on the matter, we are surprised with the small amount of sleep with which we endeavor to get along. The fact is proven beyond a doubt, that one-third of our time should be spent in sleep—quiet, restful, natural, refreshing sleep. Instead of this, many men, especially literary men, habitually starve the brain incessantly in this matter, until they induce an incurable insomnia. An irritable man, cross, pettish, disagreeable, can nearly always be put down as one who takes little sleep. A self-composed man, quiet, thoughtful, of pleasant disposition and genial nature may be known as one who is not ashamed to acknowledge that he insists upon having his full quota of sleep. Sleep is nature's great restorer, it is the best and most natural tonic to the nervous system known. Freedom from care and responsibility, a pleasant surrounding atmosphere, a good digestion, and full term of sleep, if demanded by every man would lengthen the life of the present generation from five to twenty years. It is a good plan for brain workers to take a nap after dinner. This plan is objected to by many physicians, but it is a good plan in many cases, perhaps not universally applicable, but of great value to those whose appetites and digestion must be encouraged, and who are troubled with a mild form of insomnia. These will stand their laborious work better, and will suffer less upon the loss of their regular sleep. There are some men who have stood an enormous amount of work with but very little sleep. These, it is believed, have the power to rest certain portions of the brain while other portions of the brain are active. This fact is applicable, however, to but very few men, the majority need an abundance of sleep, and the hours spent in sleep in these cases will be more than added to their term of years.—*Ex.*

